# Editorial: Current therapeutic approaches in Alzheimer's disease: the use of a second drug with anti-amyloid-beta and beyond

**DOI:** 10.3389/fnins.2024.1449365

**Published:** 2024-07-08

**Authors:** Joon W. Shim, Hyacinth I. Hyacinth, Oscar Gonzalez-Perez

**Affiliations:** ^1^Department of Biomedical Engineering, College of Engineering and Computer Sciences, Marshall University, Huntington, WV, United States; ^2^Department of Neurology and Rehabilitation Medicine, University of Cincinnati College of Medicine, Cincinnati, OH, United States; ^3^Laboratory of Neuroscience, School of Psychology, University of Colima, Colima, Mexico

**Keywords:** Alzheimer's disease, beta amyloid, monoclonal antibody, Levetiracetam, GLP-1, ABC transporter

In the continuous fight against Alzheimer's disease (AD) or Alzheimer's Disease Related Dementia (ADRD)—a formidable neurodegenerative disorder affecting millions globally—the exploration of innovative therapies is increasingly becoming multi-dimensional and dynamic. This editorial highlight the progressive strides made under the Research Topic “*Current therapeutic approaches in Alzheimer's disease: the use of a second drug with anti-amyloid-beta and beyond*,” where recent studies and reviews are pioneering beyond conventional treatment frameworks.

Among the outstanding contributions, the study led by Paul Territo's team is particularly notable, marking a substantial advancement in our molecular understanding of Alzheimer's disease. Utilizing the 5XFAD mouse model, their research published in Burton et al., delivers crucial insights into how Levetiracetam modified the metabolic networks and transcriptomic signatures within the brain. This revelation not only enriches our comprehension of the altered biochemical landscapes in Alzheimer's but also paves the way for potential early-stage metabolic interventions, as well as drug repurposing.

Expanding the boundaries of current research, Barrett et al. presented their compelling RNA-Seq analysis findings that encompass multiple species, including humans. Their focus on the GLP-1R receptor, traditionally targeted in obesity and diabetes treatments, underscores its potential viability as a therapeutic agent for Alzheimer's disease. This cross-disciplinary investigation exemplifies the translational essence of modern biomedical research, illustrating how existing therapies can be repurposed to offer new hope in combatting AD.

In the arena of systematic reviews, Li et al., have meticulously evaluated the efficacy and safety of anti-amyloid-beta agents. Their critical assessment of these agents' and their potential to delay cognitive decline offers an incisive perspective on the current therapeutic landscape. Furthermore, Doran and Sawyer, through their mini-review, delve into the risk factors associated with amyloid-related imaging abnormalities (ARIA), providing a detailed analysis essential for refining patient management strategies.

Adding to the innovative discourse, Yu et al. recent publication discusses how P-hydroxybenzaldehyde might protect against oxidative stress and β-amyloid toxicity in *Caenorhabditis elegans*, introducing potential novel antioxidant strategies in Alzheimer's disease therapy.

Finally, the review article by Loeffler on ABE transporters significantly attempted to further our understanding of amyloid-beta clearance mechanisms in the brain. These insights are vital for developing therapies aimed at enhancing the brain's inherent capability to clear amyloid, offering a supplementary route to direct pharmacological interventions (Loeffler).

These collective works not only mirror the rich diversity of ongoing Alzheimer's research but also signify a paradigm shift toward integrating genetic, molecular, and clinical insights. As we navigate the complexities of Alzheimer's disease, this holistic and innovative approach will undoubtedly be our strongest ally in forging effective treatments. This thematic collection on Alzheimer's treatment strategies showcases a firm commitment to innovation—a beacon of hope for the millions impacted by this relentless condition. The journey ahead is definitively paved with diversity in thought and unity in purpose, guiding us toward a future where Alzheimer's disease no longer steals the essence of our loved ones.

## Author contributions

JS: Conceptualization, Writing – original draft, Writing – review & editing. HH: Writing – original draft, Writing – review & editing. OG-P: Writing – original draft, Writing – review & editing.

